# Difference in presentation, outcomes, and hospital epidemiologic trend of COVID-19 among first, second, and third waves: a review of hospital records and prospective cohort study

**DOI:** 10.1097/MS9.0000000000001024

**Published:** 2023-07-06

**Authors:** Reaz Mahmud, Md. Ashikul Islam, Md. Emdadul Haque, Dewan A. Hussain, Mohammad R. Islam, Farhana B. Monayem, Mohammad M. Kamal, Hashmi Sina, Mohammad F. Islam, Ponkaj K. Datta, S.K. Jakaria B. Sayeed, Sabbir A. Dhali, Khairul Islam, Rifat H. Ratul, S.K. Md. Rubaed Hossain, Habib N. Prince, Ahmed H. Chowdhury, Kazi G.U. Ahmed, Md. Titu Miah, Md. Mujibur Rahman

**Affiliations:** a Department of Neurology, Dhaka Medical College; b National Institute of Neurosciences and Hospital; c National Institute of Chest Diseases and Hospital; d Department of Medicine, Shaheed Suhrawardy Medical College; e Sarkari Karmachari Hospital; f Department of Medicine, Dhaka Medical College; g COVID-19 Post-acute Care and Follow-up Clinic, Dhaka Medical College; h North South University; i Department of Medicine, Bangabandhu Sheikh Mujib Medical University, Dhaka, Bangladesh

**Keywords:** COVID waves, COVID-19 epidemiologic trend, COVID-19 presentation, COVID-19, post-COVID conditions

## Abstract

This study aimed to examine the differences in epidemiologic and disease aspects among patients with coronavirus disease-19 (COVID-19).

**Methods:**

The authors reviewed the hospital records between April 2020 and September 2021 and followed up on the patients for post-COVID complications.

**Findings:**

Older adult patients were predominantly affected during the third wave, and middle-aged patients were predominantly affected during the first and second waves. Men were predominantly admitted, considering the three waves, although more women were admitted in the second wave. Cough was more common in the second and third waves than in the first wave 522 (59.7%). Respiratory distress was the most common in the third wave, 251(67.1%), and least common in the first wave, 403 (46.1%). Anosmia was more common in the third wave 116 (31.2%). In the third wave, patients presenting in a critical state 23 (6.2%) and with severe disease 152 (40.8%) were more common. The hospital admission median (IQR) was longer in the first wave, 12 (8–20), than in other waves. More patients were admitted in the first wave (52%) than in the other waves, and patients received more oxygen in the third wave (75%) than in the other waves. Death occurred more commonly in the first wave (51%) than in the other waves. The positivity rate was higher in the third wave (22.8%) than in the other waves. In the third wave, the positivity rate was higher in women (24.3%) than in men. Post-COVID cough increased in the second wave, and fatigue was higher in the third wave than in the other waves. Tiredness and memory loss were greater during the second wave than in other waves.

**Conclusion:**

The authors found differences in the presentation, outcomes, and hospital epidemiologic trend of COVID-19 among the three waves.

## Introduction

Highlights

- The hospital epidemiologic trend differed in three coronavirus disease-19 waves; notably, the first wave lasted longer.
- The symptom cluster differed among the three waves.
- There were disparities in the frequencies of the positivity test and death rates.

On 31 December2019, the WHO formally reported a case of atypical pneumonia in Wuhan City, China^1^, which was later named coronavirus disease-19 (COVID-19) and caused by severe acute respiratory syndrome coronavirus 2 (SARS-CoV-2)^2^. On 11 March 2020, WHO declared the novel coronavirus (COVID-19) outbreak a global pandemic^3^.

The WHO classified the phases of the pandemic as phases 1–3 (predominantly animal infection, few human infections), phase 4 (sustained human-to-human infection), phases 5 and 6 (widespread human infection), post-peak (possibility of recurrent events), and post-pandemic (disease activity at seasonal level) (https://www.worldometers.info/coronavirus/). Most of the worst pandemics lasted for several years (https://www.who.int/influenza/resources/documents/pandemic_phase_descriptions_and_actions.pdf). After studying the transmission dynamics of SARS-CoV-2, researchers postulated that it would reach long-term circulation within the next 5 years (https://www.history.com/news/pandemics-end-plague-cholera-black-death-smallpox). Experience in the last 2 years has revealed that resurgence as ‘waves’ is common (https://www.worldometers.info/coronavirus/), and the overall pattern of the coronavirus pandemic has been a series of increased COVID-19 waves, which gradually declines. Several outbreaks of illnesses have occurred in various parts of the world. Europe entered its third phase in March 2021^4^. According to the WHO, the COVID-19 variant initially detected in the UK has spread widely in at least 27 European countries, with Denmark, Italy, Ireland, Germany, France, the Netherlands, Spain, and Portugal being the most affected (https://www.theguardian.com/world/2021/mar/22/lockdowns-return-extended-third-wave-covid-europe) and at that time the United States was likely to experience the fourth wave (https://time.com/5951490/covid-fourth-wave/). The seasonal variation in transmission, duration of immunity, degree of cross-immunity between SARS-CoV-2 and other coronaviruses, intensity, and timing of control measures^4^, and not the least viral mutation can all explain the wave and gradual decline of cases (waves of the disease) (https://www.who.int/csr/don/31-december-2020-sars-cov2-variants/en/). SARS-CoV-2 has mutated at a pace of about 1–2 mutations per month throughout the current global crisis (https://www.nature.com/articles/d41586-020-02544-6). A variant of SARS-CoV-2 with the *D614G* mutation emerged in China in late January and early February 2020 and immediately became the dominant form of the virus circulating globally (https://www.who.int/csr/don/31-december-2020-sars-cov2-variants/en/). Subsequently, several mutations were recognized as mutations of concern, naming the UK variant known as 501Y. V1, VOC 202012/01, and B.1.1.7, a South African variant known as the 501Y. V2, or B.1.351, a Brazilian variant known as 501Y. V3 or P.1 lineage^5^ (https://www.nature.com/articles/d41586-020-02544-6, https://www.who.int/csr/don/31-december-2020-sars-cov2-variants/en/) and the Indian variant B.1.617 (double mutant) (https://www.cdc.gov/coronavirus/2019-ncov/science/science-briefs/scientific-brief-emerging-variants.html). The Indian variant gave rise to India’s second wave and stressed its health system. In terms of test positivity rates and case fatality rates, the epidemiologic characteristics seem to differ from those of the first wave (https://www.who.int/docs/default-source/coronaviruse/situation reports/20210427_weekly_epi_update_37.pdf?sfvrsn=a1ab459c_5&download=true). The Bangladesh Government reported the peak of the second wave in late February 2021 and the third wave in June 2021^6^. In the second wave, the death rate increased among the young (https://www.tbsnews.net/coronavirus-chronicle/covid-19-bangladesh/covid-deaths-drop-41-lowest-39-days-242425). The African variant was responsible for more than 80% of the second wave, while the delta variant was responsible for 78% of the third wave (https://www.tbsnews.net/coronavirus-chronicle/covid-19-bangladesh/covid-deaths-drop-41-lowest-39-days-242425, https://www.icddrb.org/news-and-events/icddrb-in-the-news). Preliminary reports of various SARS-CoV-2 mutant strains show transmissibility, severity, and case mortality diversity. The initial study about the African variant reported no significant correlation between severe disease and outcomes. However, higher transmissibility was possible. The mutant virus behavior in our populations remains largely unknown. It is essential to understand the variations in how COVID-19 is presented, the resulting outcomes, and the epidemiological trends in hospitals during different waves of the pandemic. Therefore, this study aimed to systematically and scientifically observe the difference in the presentation and outcome of patients with COVID-19 between the first, second, and third waves. Additionally, we examined the positivity rate, hospital admission rate, and death rate in the outpatient department.

## Materials and methods

This study aimed to examine differences in the epidemiology of COVID-19 in the first, second, and third waves. We aimed to find out if there were any variations in the symptoms, disease severity, case fatality, length of hospital stays, frequency of patients requiring oxygen therapy, and referrals to the ICU. We reviewed the outpatient data for clinic attendance, hospital admission rate, and death in the outpatient department. We examined the virology laboratory data to observe differences in the number of tests and positivity rate with the age and sex variation. We reviewed inpatient hospital data for rates of hospital admission and death. We also assessed the differences in complications of COVID-19 in patients followed up for at least 6 months after hospital discharge during the first, second, and third waves of COVID-19. The work has been reported in line with the STROCSS (strengthening the reporting of cohort, cross-sectional and case–control studies in surgery) criteria^7^, Supplemental Digital Content 1, http://links.lww.com/MS9/A170. This study was registered at www.researchregistry.com.

### Study area and period

The study was conducted at a tertiary care hospital between 1 January 2021 and 20 June 2022.

### Study design

We reviewed the hospital records of outpatients, inpatients, and virology departments. From that patient, some sampled patients (200 patients for each wave) were prospectively followed up for the post-COVID symptoms at least 6 months after discharge. The follow-up was given by telephonic interview following a prespecified telephonic interview guide.

### Study population

The study population was the admitted patient and the patient who responded to the telephone at least 6 months after follow-up.

### Eligibility criteria

The study included patients’ records containing important demographics such as age, sex, area of residence, telephone number, complete treatment sheet, admission and discharge date, and at least a brief history. Patients more than 18 years of age, with real-time reverse transcriptase-polymerase chain reaction positive test results, irrespective of the severity of the disease, admitted between April 2020 and September 2022 were included in this study for analysis and follow-up. Patients who did not meet the study’s criteria, such as those with incomplete or inconclusive data or those outside the specified time period, were excluded from the study.

### Sample size determination

The sample size was calculated using the formula below, and the hypothesis was that there was no variation in presentation between the COVID-19 waves in Bangladesh during the pandemic:


$$n=\frac{{\left[Z_{\alpha },\sqrt{2p\left(1-p\right)},+,Z_{\beta },\sqrt{p_{1}\left(1-p_{1}\right)+p_{2}\left(1-p_{2}\right)}\right]}^{2}}{{\left(p_{1}-p_{2}\right)}^{2}}.$$

Here, *Z*_*α*_=1.96, *Z* value of standard normal distribution at 95% CI, P1=prevalence of severe infection in the first wave, P2=prevalence of severe disease in the second wave, RR=1.5 (risk ratio), P2=0.12; in one study in our country, the prevalence of severe infection was 11%^7^, P1=RR×*p*_2_=0.16, *Z*_*β*_=0.84 at 80% power, *P*=(*p*1+*p*2)÷2=0.13. Thus, the estimated sample size was 196 for each wave.

### Sampling technique and procedure

Cluster sampling methods were used to select the patients to observe the difference in the presentation and outcome of COVID-19. We compiled a list of all wards where patients with COVID-19 had been admitted. We selected one male ward and one female ward randomly. We subsequently reviewed all records that met the inclusion and exclusion criteria. We stratified the patient according to the time of the wave and tried to contact every consecutive patient for post-COVID follow-up who elapsed 6 months after discharge until we recruited 200 patients in each wave. For those who lost the follow-up, we excluded them from the study.

### Study variables

The variables included in the study were:

#### Demographic variable

Age of the respondents in years and sex.

#### Presenting features of COVID-19

Fever, cough, respiratory distress, sore throat, nausea, vomiting, diarrhea, and body ache.

#### Comorbid conditions

We observed pre-existing health conditions, particularly hypertension, diabetes, ischemic heart disease, pulmonary diseases such as bronchial asthma or COPD, and renal disease before being infected with COVID-19.

#### Investigations in COVID-19 state

Total blood count, CRP, D-dimer, creatinine, and ferritin levels.

#### COVID-19 severity

The severity of COVID-19 is contingent upon the symptoms one undergoes, which can range from being asymptomatic to experiencing life-threatening conditions (asymptomatic, mild, moderate, severe, or critical).

#### Duration

Duration between symptom onset and hospital admission, length of hospital stay.

#### Post-COVID-19 conditions

Fatigue, cough, respiratory distress, insomnia, etc.

#### Outcome-related questions

Discharged and death, oxygen requirement, ICU referral.

#### Functional impact

We examined functional implications in the post-COVID state for at least 6 months for overall functional status, fatigue score, and depression score.

#### Operational definition

Confirmed COVID-19 and its different severities were defined according to the WHO and National Guidelines^8,9^.

#### Case fatality rate


$$\mathrm{CFR}=\frac{\mathrm{NUMBER OF TOTAL DEATH DURING THE SAME PERIOD IN THE SAME WARD}}{\mathrm{NUMBER OF TOTAL ADMISSION IN THE DEFINED PERIOD IN THE SELECTED WARD}}\times 100.$$

#### Duration of hospital stay

The total period from the day of admission to the day of discharge.

#### Waves of infection

The first, second, and third waves extend between April 2020 and January 2020, February 2021 and May 2021, and June 2021 and September 2021, respectively^10,11^.

#### Post-COVID-19 condition: according to the Center for Disease Control, USA^12^

Post-COVID conditions include a wide range of new, returning, or ongoing health problems that people experience after contracting the SARS-CoV-2 causing COVID-19.

#### Post-COVID fatigue

The diagnostic criteria for post-COVID-19 fatigue were developed according to the United States Institute of Medicine symptom criteria^13^.

The Chalder Fatigue Scale^14^, the Karnofsky Performance Status Scale^15^, and the Depression Scale were used to assess fatigue severity, overall functional status, and depression^16^.

### Data collection instrument

For patients admitted to the hospital, data were collected on a data collection sheet. A telephone directory was used for post-COVID-19 follow-up. We collected data from reports of the outpatient, inpatient, and virology departments.

### Data collection procedure

We collected all patients’ files from the hospital record unit in the selected ward. Each file was evaluated by one data collector and researcher. The completed data collecting sheet contains a list of all available information. We obtained no specific procedure for missing data and excluded files that did not contain essential information, such as patient demographics, admission date, and discharge date. We collected the phone number from the hospital records and interviewed the patients for post-COVID-19 complications at least 6 months after recovery. We collected data from the outpatient, inpatient, and virology departments using the Excel datasheet, which contains the patient’s name, age, sex, date of attendance, outcome, admission, death, discharge, positive or negative status, etc.

### Data quality control

Each datasheet was reviewed by two researchers; disagreements were resolved through discussion. We removed responses where respondents provided contradictory answers. Because each patient was coded and had a specified hospital record number, there was no scope for double responses.

### Data processing and analysis

R (v4.1.1) was used to process data. We performed a network analysis with a tidy verse and q graph. Qualitative data with normal distribution were expressed as means (SD), while non-normal data were expressed as medians (IQR). We divided the respondents into groups of first, second, and third waves. We used the *χ* ^2^-test to calculate qualitative data, one-way ANOVA to calculate normally distributed quantitative data, and Kruskal–Wallis test to calculate skewed quantitative data. As needed, we used a post hoc analysis with Bonferroni adjustment, Dunn test, and Tukey’s test. We determined epidemiological trends in the EXECL sheet. The *P*-value for statistical significance was set at <0.05. We did not impute any missing values, and they were included in the analysis.

### Ethical consideration

The Institutional Ethical Committee of the respected Hospital approved this study (ERC-DMC/ECC/2021/55). As we mainly reviewed the hospital data, there was no need for written consent. The patients provided verbal consent for post-COVID follow-up.

## Results

We reviewed 1766 patient records and included 1595 patients’ data for analysis. From these patients, we followed 600 patients over the telephone for post-COVID-19 complications. Between April 2020 and September 2021, 38 578 patients visited the outpatient department, 24 501 patients were admitted, and 57 857 were tested at the virology department (Supplement 1, Supplemental Digital Content 2, http://links.lww.com/MS9/A171).

The mean age (SD) of the hospital-admitted patients was 47.9 (15.67) years, with patients in the second and third waves being higher than those in the first wave (*P*<0.001).

Men were predominantly admitted to 968 (60.68%). However, in the second wave, the number of women, 191 (54.7%), was higher.

Cough was more prevalent in the second 288 (65.5%) and third waves 290 (78%) than in the first wave 522 (59.7%). Running nose was most prevalent in the first wave, 143 (16.4%), and least prevalent in the second wave, 16 (4.6%). Respiratory distress was the most prevalent in the third wave, 251 (67.1%), and the least prevalent in the first wave, 403 (46.1%). Sore throat occurred more prevalent in the first wave 198 (22.7%) than in the other waves. Diarrhea 60 (16.1) and vomiting 59 (15.9%) were the most prevalent in the third wave. Anosmia was more prevalent in the third wave 116 (31.2%) than in other waves and less frequently in the second wave 27 (7.8%) than in other waves. However, headache was more prevalent in the third 82 (22.5%) and first waves 145 (16.6%) than in the second wave. Body aches occurred more commonly in the second 87 (25%) and third 92 (24.6%) waves than in the first wave (Table 1).

**Table 1:** Demographic characteristics and comorbidity of the admitted patients in the three waves

Variable	Categories	Total patient (*n*=1595), *N* (%)	First wave^a^ (*n*=874), *N* (%)	Second wave^b^ (*n*=349), *N* (%)	Third wave^c^ (*n*=372), *N* (%)	*P*
Age (*n*=1595), mean (SD)		47.91 (15.67)	43.82 (14.31)	50.79 (15.95)	54.62 (15.61)	<0.001
Age (*n*=1595)	<40 years	512 (32.1)	364 (41.6)	84 (24.1)	64 (17.2)	<0.001
	40–60 years	652 (40.8)	366 (41.9)	139 (39.8)	147 (39.5)	
	60+ years	431 (27.02)	144 (16.8)	126 (36.1)	161 (43.3)	
Sex (*n*=1595)	Female	627 (39.31)	283 (32.4)	191 (54.7)	153 (41.0)	<0.001
	Male	968 (60.68)	590 (67.6)	158 (45.3)	220 (59.0)	
Fever (*n*=1595)	Present	1366 (85.6)	738 (84.4)	296 (85.1)	332 (89.0)	0.1
Cough (*n*=1595)	Present	1040 (65.2)	522 (59.7)	228 (65.5)	290 (78.0)	<0.001
Running nose (*n*=1595)	Present	206 (12.9)	143 (16.4)	16 (4.6)	47 (12.6)	<0.001
Respiratory distress (*n*=1595)	Present	848 (53.1)	403 (46.1)	194 (55.7)	251 (67.1)	<0.001
Sore throat (*n*=1595)	Present	278 (17.4)	198 (22.7)	33 (9.5)	47 (12.6)	<0.001
Hoarseness of voice (*n*=1595)	Present	24 (1.5)	11 (1.3)	5 (1.4)	8 (2.1)	0.50
Chest pain(*n*=1595)	Present	107 (6.7)	52 (6.0)	19 (5.4)	36 (9.6)	0.03
Diarrhea (*n*=1595)	Present	152 (9.5)	74 (8.5)	18 (5.2)	60 (16.1)	<0.001
Vomiting (*n*=1595)	Present	96 (6.1)	29 (3.3)	8 (2.3)	59 (15.9)	<0.001
Anorexia (*n*=1594)	Present	274 (17.1)	123 (14.1)	38 (11.0)	113 (30.2)	<0.001
Anosmia (*n*=1593)	Present	301 (18.8)	158 (18.1)	27 (7.8)	116 (31.2)	<0.001
Headache (*n*=1583)	Present	252 (15.7)	145 (16.6)	25 (7.2)	82 (22.5)	<0.001
Lethargy (*n*=1595)	Present	436 (27.3)	231 (26.4)	106 (30.4)	99 (26.5)	0.35
Body ache (*n*=1595)	Present	286 (17.9)	107 (12.2)	87 (25.0)	92 (24.6)	<0.001
Presenting severity^d^ (*n*=1595)	Critical^e^	25 (1.5)	0 (0.0)	2 (0.6)	23 (6.2)	<0.001
	Mild^f^	747 (29.7)	422 (48.3)	216 (61.9)	109 (29.2)	
	Moderate^g^	648 (40.6)	443 (50.7)	116 (33.2)	89 (23.9)	
	Severe^h^	175 (10.9)	8 (0.9)	15 (4.3)	152 (40.8)	
Comorbidities						
Diabetes (*n*=1151)	Present	531 (33.2)	216 (28.4)	136 (38.8)	179 (48.1)	<0.001
Hypertension (*n*=1154)	Present	532 (33.3)	182 (24.5)	142 (40.6)	208 (55.9)	<0.001
Asthma (*n*=931)	Present	114 (7.1)	57 (8.0)	30 (8.5)	27 (16.0)	<0.001
IHD (*n*=930)	Present	123 (7.7)	29 (4.1)	31 (8.8)	63 (16.9)	<0.001
Renal disease (*n*= 926)	Present	103 (6.4)	24 (3.4)	32 (9.1)	47 (12.6)	<0.001

^a^ From April 2020 to January 2020.

^b^ From February 2021 to May 2021.

^c^ From June 2021 to September 2021.

^d^ The severity of the patient at the time of hospital admission.

^e^ Critical: Patient requiring high flow oxygen or mechanical ventilation.

^f^ Mild: Patients having mild symptoms of upper respiratory tract viral infection, including mild fever, cough (dry), sore throat, nasal congestion, malaise, headache, muscle pain, anosmia, or malaise.

^g^ Moderate: the patient has respiratory symptoms such as cough and shortness of breath are present without signs of severe pneumonia (tachypnea >30 breaths/min and hypoxia: oxygen saturation <90 on room air).

^h^ Severe: Patients with severe pneumonia require supplemental oxygen through nasal canal, facemask, or face mask with reservoir.

Patients that presented in a critical condition 23 (6.2%) and severe disease 152 (40.8%) were more prevalent in the third wave than in other waves. In the second wave, most cases were mild 216 (61.9%) (Table 1).

Different comorbid conditions, notably diabetes, hypertension, asthma, ischemic heart disease, and renal disease, differed in the three waves (Table 1).

### Symptoms cluster in COVID-19

**Figure 1:**
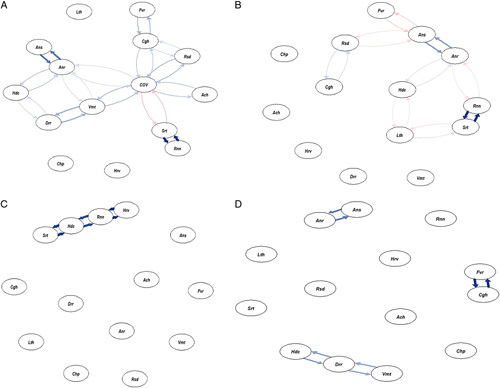
Symptoms clusters in coronavirus disease-19 (COVID-19). (A) According to general clusters of symptoms, we found three clusters of symptoms among the admitted patients with COVID-19: (1) fever, cough, and respiratory distress; (2) anosmia, headache, and anorexia; (3) sore throat and running nose. (B) In the first wave, we found three clusters: (1) respiratory distress and cough; (2) anosmia anorexia; and (3) running nose and sore throat. (C) In the second wave, we found a cluster headache, running nose, sore throat, and hoarseness of voice. (D) In the third wave, we found three clusters: (1) anorexia and anosmia; (2) fever and cough; and (3) headache, diarrhea, and vomiting. We did the network analysis with tidy verse and q graph using R (v4.1.1). Ach, body ache; Anr, anorexia; Ans, anosmia; Cgh, cough; Chp, chest pain; cov, COVID-19; Drr, diarrhea; Fvr, fever; Hdc, headache; Hrv, hoarseness of voice; Lth, lethargy; Rnn, running nose; Rsd, respiratory distress; Srt, sore throat; Vmt, vomiting.

We found three clusters of symptoms among the admitted COVID patients: (1) fever, cough, and respiratory distress; (2) anosmia, headache, and anorexia; (3) sore throat and running nose. The symptom clusters differed for the three waves. In the first wave, we found three clusters: (1) respiratory distress and cough; (2) anosmia and anorexia; (3) running nose and sore throat. In the second wave, we found a cluster of headaches, running nose, sore throat, and hoarseness of voice. In the third wave, we found three clusters: (1) anorexia and anosmia; (2) fever and cough; and (3) headache, diarrhea, and vomiting (Fig. 1).

The white blood cell count (10^9^/l) was high in the second wave, 18.65 (14.5–24), and low in the third wave. The platelet count (10^9^/l) was low in the third wave 78 (68–86). CRP (mg/dl) was much higher in the third wave 39 (13–85) and D-dimer (mg/L) 1.52 (0.63–3.56), and ferritin ((μg/l) was high in the second wave 1468 (973–2050) (Table 2).

**Table 2:** Investigation profile of the admitted patients in the three waves

Variable	Total patient (*N*)	First wave, median (IQR)	Second wave, median (IQR)	Third wave, median (IQR)	*P*
WBC (10^9^/l)	607	8.50 (6.2–11.72)	18.65 (14.55–24.00)	5.95 (1.28–10.23)	<0.001
Neutrophil (%)	595	71.70 (60.92–83.00)	83.00 (79.0–87.0)	78.00 (68.0–86.0)	<0.001
Lymphocyte (%)	595	21.75 (12.88–30.68)	12.00 (10.70–16.0)	18.00 (10.0–27.0)	<0.001
Platelet count (10^9^/l)	575	247.00 (189.5–324.0)	126.00 (29.9–276.00)	135.00 (31.00–250.00)	<0.001
CRP (mg/dl)	438	12.00 (6.0–34.77)	18.00 (8.68–68.50)	39.00 (13.0–85.0)	<0.001
Creatinine (mg/dl)	547	0.96 (0.80–1.12)	1.180 (0.95–6.62)	1.14 (0.90–1.40)	<0.001
D-dimer (mg/l)	458	0.47 (0.27–1.05)	1.52 (0.62–3.56)	0.79 (0.40–1.80)	<0.001
Ferritin (μg/l)	369	297.00 (122.0–642.0)	1468.00 (973.5–2050.0)	441.00 (235.5–1000.0)	0.002
Serum sodium (mmol/l)	191	–	138.00 (135.0–140.8)	135.00 (132.0–138.0)	0.121

During the hospital stay, the severity of the patient’s severity changed, and in the third wave, 72 (19.3%) critical and 145 (38.8%) severe diseases emerged. Mild-to-moderate illness was more common in the first and second waves than in the third wave (Table 3). The duration of hospital stay was longer in the first wave 12 (8–20) (Table 3).

**Table 3:** Outcome of the admitted patient in three waves

Variable	Categories	Total patient, *N*	First wave, *N* (%)	Second wave, *N* (%)	Third wave, *N* (%)	*P*
Ultimate severity	Critical	107	32 (29.9)	3 (2.8)	72 (67.3)	<0.001
	Mild	627	365 (58.2)	183 (29.2)	79 (12.6)	
	Moderate	589	383 (65.0)	128 (21.7)	78 (13.2)	
	Severe	271	94 (34.7)	32 (11.8)	145 (53.5)	
Duration of hospital stay	—	—	13.0 (8.0−20.0)	12.0 (8.0−12.0)	7.0 (4.0−11.0)	<0.001
Interval symptom onset and admission	—	—	5.0 (4.0−6.0)	5.0 (5.0−6.0)	5.0 (4.0−6.0)	<0.001

Confirmed COVID-19 cases were higher (15.7%) in the second wave than in the other waves; the admission rate of 73.7% and the death rate of 1.3% were higher in the second wave than in other waves. The proportion of confirmed COVID-19 cases was higher among women in all waves. In the third wave, the admission rate among women was higher than among men. The number of positive cases was higher in those below 40 years in the first wave and above 60 years in the third wave. The admission rate of patients below 40 years was lower in the third wave. In the second wave, the admission rate among the 40–60-year age group and above 60 years age group was higher than in other waves. In the second wave, the death rate was higher among men, 1.4%, than among women, 1.3%. In the second wave, the death rate was high in the 40–60-year age group and above the 60-year age group. The admission rate was higher in patients aged above 60 years in all three waves.

Death was more common (51% of total death) in the first wave.

The patient was tested more frequently in the first and third waves than in the second wave. The positivity was high in the third wave, at 22.8%. In the third wave, the positivity rate was high among women, 24.3%. The incidence rate of patients above 60 years was high in the first and third waves, at 27.3% (Table 4).

**Table 4:** Trend of outpatient, inpatient, and virology department consultation in three waves

Trait	Total	First wave	Second wave	Third wave	*P*
Outpatient department					
Total attended	38 578	19 890 (51.6)	7347 (19.0)	11 341 (29.4)	
Positive	5584	3134 (15.7%)	896 (12.1%)	1554 (13.7%)	<0.001
Admitted	25 721	13 650 (68.6%)	5419 (73.7%)	6652 (58.6%)	<0.001
Death	329	127 (0.6%)	105 (1.4%)	97 (0.8%)	
Admission rate (%)	81.3	80.6	84.4	80.1	
Sex					
Male					
Total attended	22 595	12 274 (54.3)	4341 (19.2)	5980 (26.5)	
Positive	3201	1895 (15.4%)	518 (11.9%)	788 (13.1%)	<0.001
Admitted	15133	8541 (69.5%)	3218 (74.1%)	3374 (56.4%)	<0.001
Death	208	86 (0.7%)	64 (1.4%)	58 (0.9)	
Admission rate (%)	81.6	81.2	84.7	80.0	
Female					
Total attended	15981	7614 (47.6)	3006 (18.8)	5361 (33.5)	
Positive	2383	1239 (16.2%)	378 (12.5%)	766 (14.2)	<0.001
Admitted	10586	5107 (67%)	2201 (73.2%)	3278 (61.1%)	<0.001
Death	121	41 (0.5%)	41 (1.3%)	39 (0.6%)	
Admission rate (%)	80.9	80.0	84.0	80.3	
Age group					
<40 years					
Total attended	14 264	7297 (51.2)	2354 (16.5)	4613 (32.3)	
Positive	1623	1021 (13.9)	221 (9.3)	381 (8.2)	<0.001
Admitted	6266	3547 (48.6)	1162 (49.3)	1557 (33.7)	<0.001
Death	49	20 (0.2)	13 (0.5)	16 (0.3)	
Admission rate (%)	79.0	77.3	83.2	79.7	
40–60 years					
Total attended	12 759	6920 (54.2)	2457 (19.3)	3382 (26.5)	
Positive	2097	1177 (17)	356 (14.4)	564 (16.6)	<0.001
Admitted	9314	5099 (73.6)	1948 (79.2)	2267 (67)	<0.001
Death	121	42 (0.6)	45 (1.8)	34 (1)	
Admission rate (%)	80.8	80.7	82.9	79.1	
60+ years					
Total attended	11 490	5645 (49.1)	2516 (21.9)	3329 (29.0)	
Positive	1852	929 (16.4)	316 (12.5)	607 (18.2)	<0.001
Admitted	10101	4979 (88.2)	2300 (91.4)	2822 (84.7)	<0.001
Death	153	64 (1.1)	47 (1.8)	42 (1.2)	
Admission rate (%)	83.4	83.4	86.4	81.3	
Inpatient consultation					
Total admission	24 501	12 797 (52.2)	5317 (21.7)	6387 (26.1)	0.0004
Oxygen support	14 198	6865 (48.4)	3332 (23.4)	4001 (28.2)	<0.0001
Percentage of the patient getting oxygen		61.8%	69.43%	75.29%	<0.0001
ICU admission	4030	3442 (26.8%)	588 (11%)	–	<0.0001
Death	5057	2584 (20%)	1019 (19%)	1454 (22.7%)	<0.0001
Virology department					
Total test	57 857	26 708 (46.1)	13 214 (22.8)	17 935 (31.0)	
Positive	12 870	5962 (46.3)	2814 (21.9)	4094 (31.8)	<0.001
Positivity rate (%)	22.2%	22.3	21.3	22.8	
Sex					
Male					
Total test	32 517	15 146 (46.6)	7591 (23.3)	9780 (30.1)	
Positive	7082	3416 (48.2)	1553 (21.9)	2113 (29.8)	<0.001
Positivity rate (%)	21.8	22.6	20.5	21.6	
Female					
Total test	25 312	11 542 (45.6)	5619 (22.2)	8151 (32.2)	
Positive	5782	2542 (44.0)	1260 (21.8)	1980 (34.2)	<0.001
Positivity rate (%)	22.8	22.0	22.4	24.3	
Age group					
<40 years					
Total test	27 055	13 408 (49.6)	5291 (19.6)	8356 (30.9)	
Positive	5555	2721 (49.0)	1063 (19.1)	1771 (31.9)	<0.001
Positivity rate (%)	20.5	20.3	20.1	21.2	
40–60 years					
Total test	13033	6215 (47.7)	2570 (19.7)	4248 (32.6)	
Positive	3148	1538 (48.9)	582 (18.5)	1028 (32.7)	<0.001
Positivity rate (%)	24.2	24.7	22.6	24.2	
>60 years					
Total test	8210	3477 (42.4)	1678 (20.4)	3055 (37.2)	
Positive	2114	911 (43.1)	369 (17.5)	834 (39.5)	<0.001
Positivity rate (%)	25.7	26.2	22.0	27.3	

The daily COVID-19 infection rate was higher in the third wave, and the duration of the second wave was short. The first wave lasted longer than the other wave, with an elevated and gradual infection rate decline.

**Figure 2:**
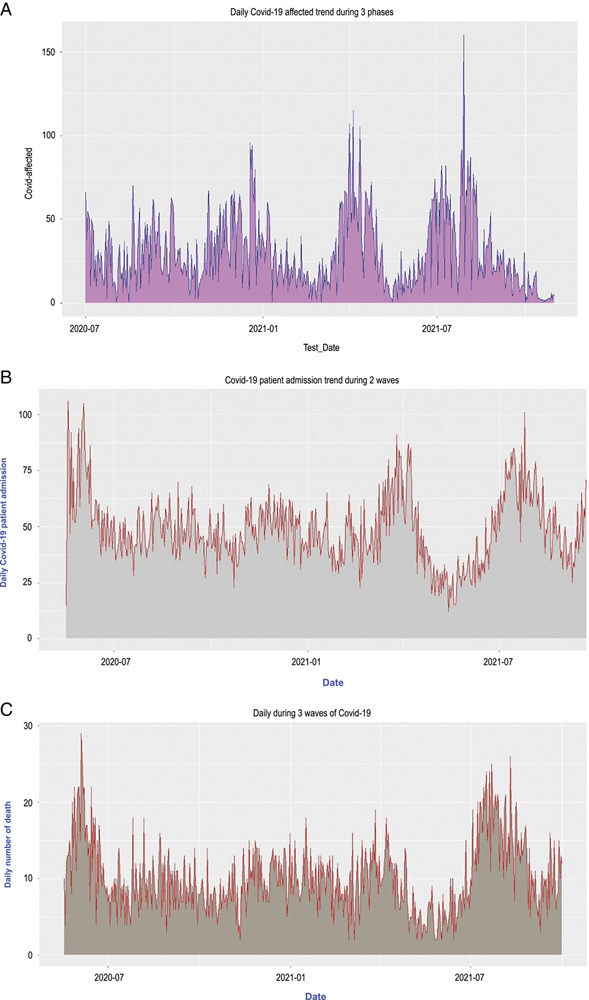
Epidemiologic trend of a number of test positivity, hospital admission, and death in three waves. (A) Test positivity – the first surge remained longer and the second surge was the narrowest. The daily number of positive cases was high in the second and third surges. (B) Daily admission – number of daily admissions was highest in the initial part of the first wave and third wave. (C) Daily death – number of daily deaths was highest in the initial part of the first wave and third wave (the first, second, and third waves extend from April 2020 to January 2020, February 2021 to May 2021, and June 2021 to September 2021, respectively).

The rate of testing was equal for all three waves. The positivity rate was higher during the third than in the first and second waves (Fig. 2, Supplement 2, Supplemental Digital Content 3, http://links.lww.com/MS9/A172).

**Figure 3:**
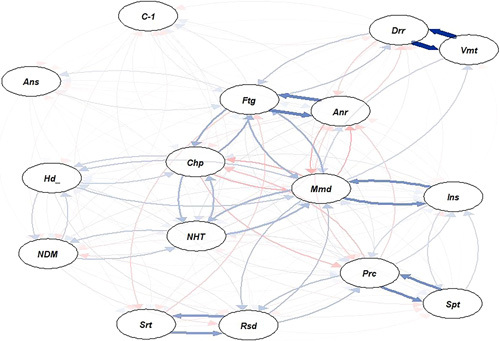
Symptom cluster in post-COVID state. There were five cluster of symptoms in the post-COVID state: (1) diarrhea and vomiting; (2) fatigue and anorexia; (3) memory loss and insomnia; (4) persistent cough and sputum; (5) sore throat and respiratory distress. We did the network analysis with tidy verse and q graph using R (v4.1.1). Anr, anorexia; Ans, anosmia; Chp, chest pain; cov, COVID-19; Drr, diarrhea; Ftg, fatigue; Hdc, headache; Mmd, memory disturbance; NDM, new onset diabetes; NHT, new onset hypertension; Ptc, persistent cough; Rsd, respiratory distress; Spt, sputum; Srt, sore throat; Vmt, vomiting.

The percentage of patients that developed post-COVID complications was equal in all three waves. However, patients in the second wave developed more post-COVID cough and, memory disturbance, depression, and patients in the third wave developed more fatigue than those in the other waves (Supplement 3, Supplemental Digital Content 4, http://links.lww.com/MS9/A173). The functional status of the patients was similar in the three waves. We found five symptom clusters. However, they did not differ among the three waves (Fig. 3).

## Discussion

This study compared the first, second, and third COVID-19 waves in terms of presentation, outcomes, and hospital epidemiological trends. The first wave lasted longer; however, the second wave lasted for a short period. We found age and sex differences in the admission rates in the three waves. Cough, runny nose, respiratory distress, diarrhea, vomiting, and anosmia were the most common symptoms in the third wave. The symptom cluster differed among the three waves. Disease severity, duration of hospital stays, and severity markers differed. We found disparities in the frequencies of the positivity test and death rates. The three waves had similar post-COVID complications.

This study was conducted at the tertiary center of Dhaka. The treatment facility differs from center to center and region to region; therefore, we cannot generalize the findings across the nation and globally.

During replication, a mutation in the genetic code of SARS-CoV-2 leads to the development of a new variant. The behaviors of variants, such as virulence and transmissibility, differ^17^. WHO named the variant to be monitored with the Greek alphabet and named from alpha to zeta (https://www.cdc.gov/coronavirus/2019-ncov/variants/variant-classifications). In this country, until September 2021, alpha, beta, and delta variants were dominant at different times creating three waves of COVID-19; the first wave was caused by alpha, the second wave was caused by beta, and the third wave was caused by delta variants^18^. Epidemiological and disease characteristics differ in various studies^6,18,19^.

In our country, the first wave lasted ~8 months, the second wave ~2 months, and the third wave ~3 months. This was due to the variations’ transmissibility; delta was 63–167% more transmissible than alpha^20^. Therefore, the most susceptible people became infected faster, replacing existing variants. Delta and beta variants spread quickly in our country, and delta quickly became the dominant variant. Omicron was 2.8 times more transmissible than Delta, thus replacing Delta in a short period^21^.

In this study, we found a variation in the demographic patterns across the three variants.

Because of their increased mobility, the younger age group became infected in large numbers^22^. Immune senescence and various comorbidities could explain the older adults’ admission. The viral receptors ACE2 and CD26 are more expressed in senescent cells, which explains why older adults are more susceptible to infection^23^. The virus affinity for the receptor varies among the variants. This explains the age group variations among variants^24^.

Men had a higher rate of incidence of COVID-19 due to the altered immunologic response, associated comorbidity, hormonal differences, and smoking habits^23^. Angiotensin-converting enzyme (ACE) receptor expression is higher in women because its genetic loci are on the X chromosome^25^. The *TMPRSS2* gene is located on chromosome 21q22.3^26^. Some variants have a higher affinity for TMPRSS than for ACE II. This might explain the sex differences in the expression of the various COVID-19 variants. Again, increased testosterone levels may increase the probability of microthrombi formation^27^, which is the underlying pathophysiology of severe COVID-19.

The duration of hospital admission was longer in the first wave. In the first wave, most patients were admitted because of isolation, and their release was determined by the time of PCR negativity. ICU admission was higher in the first wave due to the fear of an unnecessary patient transfer to the ICU. The death rate was higher in the third wave than in the other waves.

The different affinities of the viruses can explain the heterogeneous presentation and clustering of various variants of the receptor. The virus’s ability to escape immunity^28^ can explain the mutant strain’s varied presentation and severity.

Several recently published studies have confirmed variations in the demography, severity, and mortality rates during different COVID-19 waves^18,19,29–31^.

The three waves had slightly different post-COVID-19 complications. Further studies on its pathogenesis and immunological response are needed.

COVID-19 patients’ presentation, outcomes, and epidemiologic trends have demonstrated remarkable variation across different waves, as confirmed by multiple studies conducted in various countries. Our study strongly supports this observation, and a solid pathophysiological basis exists for these differences.

There are some limitations to our study. First, it was conducted at a single center, and not all patients underwent genotyping due to practical limitations. Second, since it was a retrograde study and involved a review of documents, some information may need to be included.

In each of the three waves, COVID-19 occurred differently. A mutant strain of the SARS-CoV-2 was most likely the cause of these differences. Therefore, we recommend the following:

- There should be genetic surveillance for the identification of the specific variant of interest.
- At the onset of each wave, intensive epidemiologic surveillance should be conducted.
- There should be diversity in each wave’s strategy for disease control planning, treatment, and hospital management.

## Conclusion

We found differences in presentation, outcomes, and hospital epidemiologic trend of COVID-19 among the first, second, and third waves. So genetic surveillance is essential. In the future prospective multicenter research is warranted.

## Ethical approval

The Institutional Ethical Committee of Dhaka Medical College approved the study (ERC-DMC/ECC/2021/55).

## Patient consent

As we mainly reviewed hospital data, there was no scope for written consent. We took written or verbal consent from the patient for post-COVID follow-up.

## Sources of funding

This research received grant from the Director General of Health Services, Government of the peoples’ republic of Bangladesh.

## Conflicts of interest disclosure

The authors declare that there are no conflicts of interest.

## Research registration unique identifying number (UIN)

This study was registered at www.researchregistry.com.The research registry UIN is researchregistry8714.

## Data availability statement

The data will be within the paper and uploaded as supplementary files.

## Provenance and peer review

Not commissioned, externally peer-reviewed.

## Permission to reproduce material from other sources

Not applicable.

## Supplementary Material
