# Supplementary figures and images for: Difference in presentation, outcomes, and hospital epidemiologic trend of COVID-19 among first, second, and third waves: a review of hospital records and prospective cohort study
Source: Ann Med Surg (Lond). 2023 Jul 6;85(8):3816–26. doi: 10.1097/MS9.0000000000001024 (PMC10405996; doi:10.1097/MS9.0000000000001024)

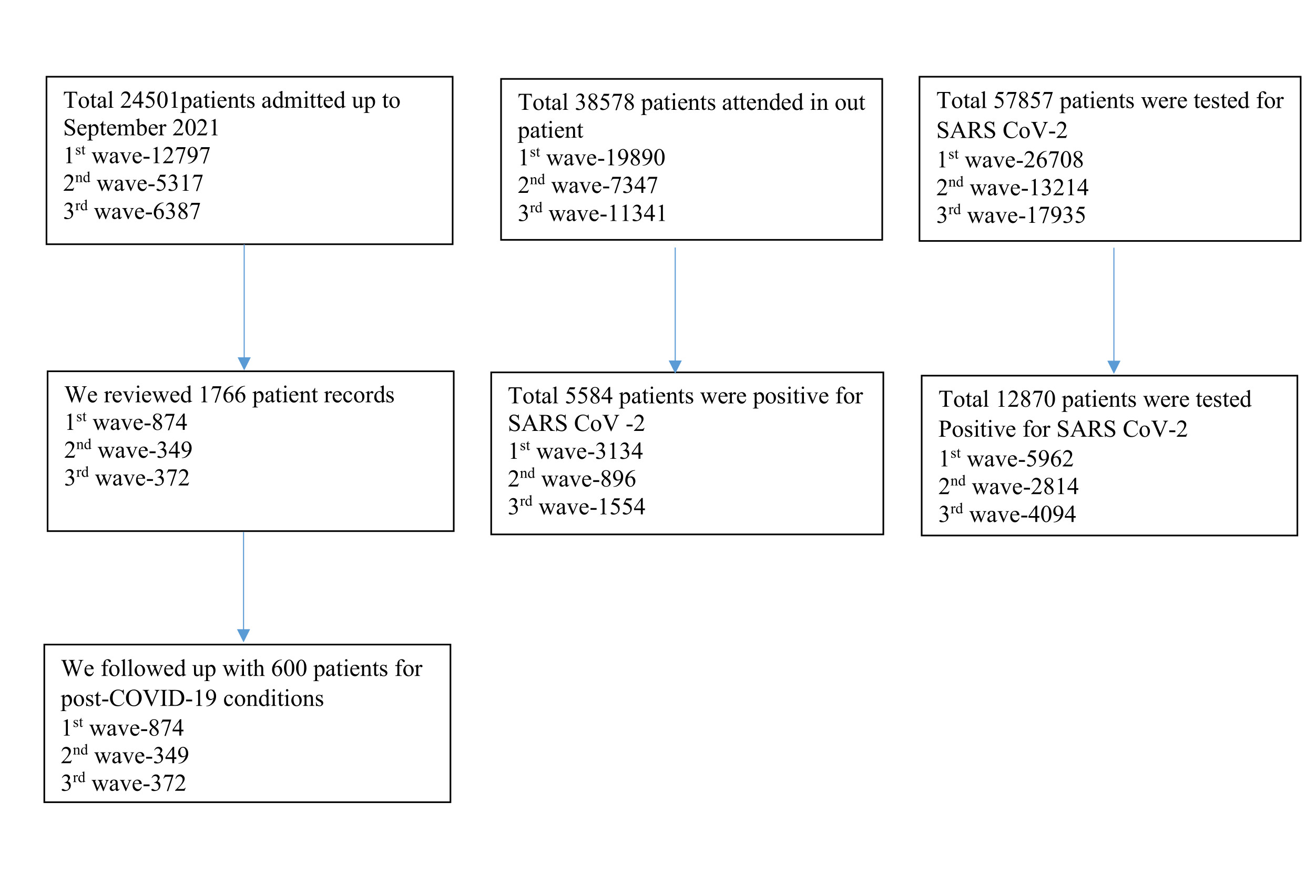

Supplement: Supplementary file 2 [file ms9-85-3816-s002.jpg]

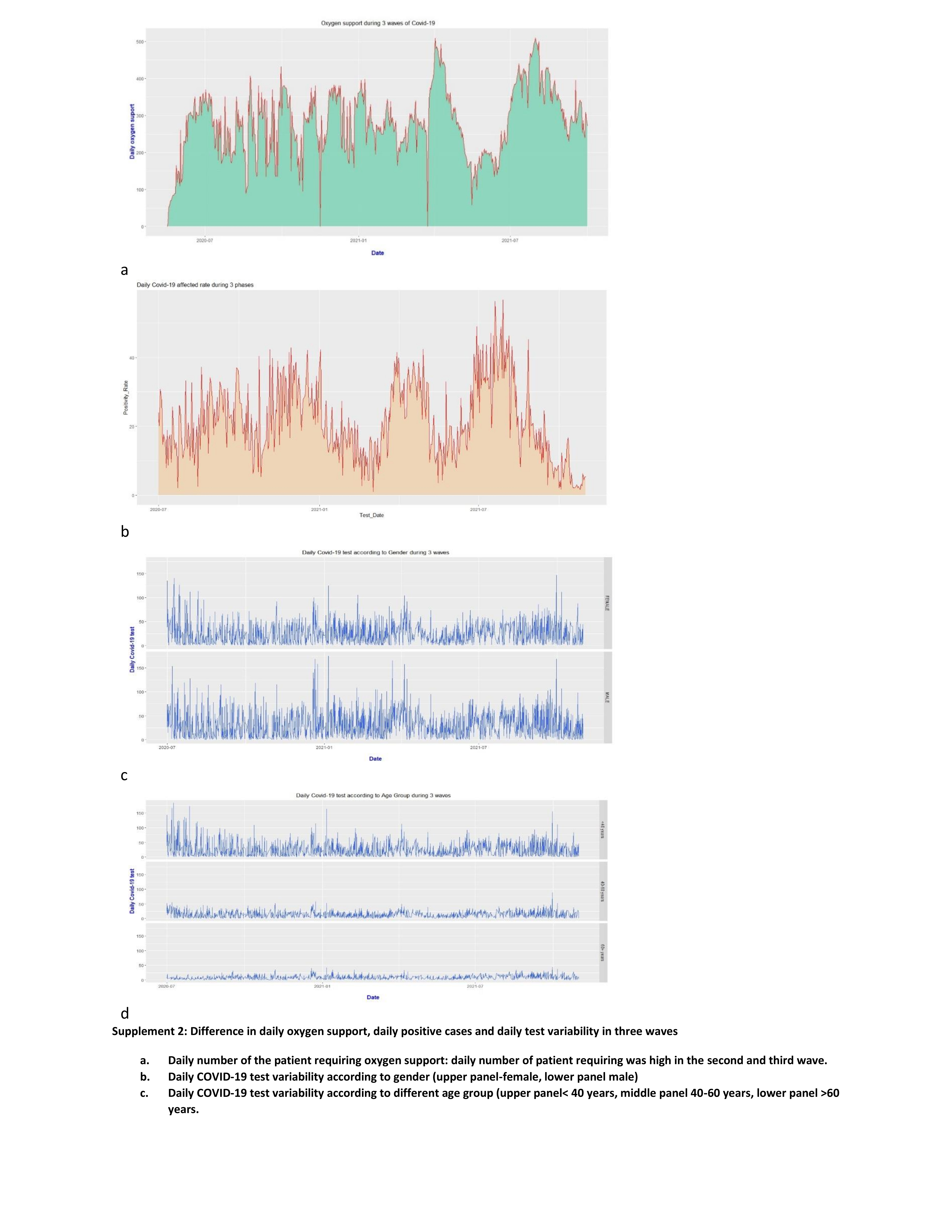

Supplement: Supplementary file 3 [file ms9-85-3816-s003.jpg]
