# Supplementary material for: Difference in presentation, outcomes, and hospital epidemiologic trend of COVID-19 among first, second, and third waves: a review of hospital records and prospective cohort study
Source: Ann Med Surg (Lond). 2023 Jul 6;85(8):3816–26. doi: 10.1097/MS9.0000000000001024 (PMC10405996; doi:10.1097/MS9.0000000000001024)
Supplement: Supplementary file 4 [file ms9-85-3816-s004.docx]

**Supplement 3: post COVID -19 complications of the three waves**

| Trait | Total patient  600 | First wave  200 | Second wave  200 | Third wave  200 | P value |
| --- | --- | --- | --- | --- | --- |
| Age, Mean (SD) | 45.2(15.8) | 45.5(16.3) | 43.6(14.3) | 46.5(16.5) | 0.19 |
| Sex, Male n (%) | 337(56.2) | 123(61.5) | 97(48.5) | 117(58.5) | 0.08 |
| Post COVID-19 conditions | | | | | |
| Number of post-covid conditions | | | | | 0.24 |
| None | 40(6.7) | 17(8.5) | 12(6) | 11(5.5) |  |
| Single | 93(15.5) | 38(19) | 30(15.1) | 25(12.5) |  |
| Multiple | 466(77.7) | 145(72.5) | 157(78.9) | 164(82) |  |
| Post COVID19 Symptoms | | | | | |
| Feverish feeling | 16(2.7) | 6(3) | 4(2) | 6(3) | 0.7 |
| Fatigue | 421(70.2) | 126(63) | 144(72) | 151(75.5) | 0.03 |
| Cough | 127(21.2) | 35(17.6) | 58(29) | 34(17) | 0.004 |
| Respiratory distress | 40(6.7) | 14(7) | 15(7.5) | 11(5.5) | 0.7 |
| Hoarseness of voice | 16(2.7) | 9(4.5) | 2(1) | 5(2.5) | 0.09 |
| Chest pain | 142(23.7) | 44(22.1) | 40(20.1) | 58(29) | 0.09 |
| Anorexia | 135(22.5) | 40(20) | 39(19.5) | 56(28) | 0.07 |
| Anosmia | 16(2.7) | 7(3.5) | 4(2) | 5(2.5) | 0.6 |
| Headache | 146(24.3) | 46(23) | 49(24.5) | 51(25.5) | 0.08 |
| Memory disturbance | 152(25.4) | 46(23) | 64(32) | 42(21.1) | 0.03 |
| Insomnia | 73(12.2) | 20(10) | 29(14.5) | 24(11) | 0.38 |
| Hypersomnia | 4(0.7) | 2(1) | 2(1) |  | 0.36 |
| Sleep pattern alteration | 6(1) | 2(1) | 2(1) | 2(1) | <0.99 |
| Arthralgia | 69(11.5) | 25(12.6) | 28(14) | 16(8) | 0.15 |
| Confusion | 29(4.8) | 5(2.5) | 12(6) | 12(6) | 0.18 |
| New onset hypertension | 15(2.5) | 2(1) | 7(3.5) | 6(3) | 0.23 |
| New onset Diabetes | 23(3.8) | 8(4) | 10(5) | 5(2.5) | 0.42 |
| Fatigue score, mean (SD) | 4.7(2.9) | 3.1(2.9) | 4.2(3.3) | 3.9(3.1) | 0.001 |
| Functional status, mean (SD) | 90.3(7.4) | 90.9(7.7) | 90(7.6) | 90(6.9) | 0.35 |
| Depression | 143(25.2) | 58(40.6) | 49(34.3) | 36(25.2) | 0.03 |
